# Relative multi-beneficial effect of MOs on plant health of chickpea (*Cicer arietinum* L. var. PG-186)

**DOI:** 10.3389/fmicb.2024.1452553

**Published:** 2024-08-27

**Authors:** Supriya Tomer, Priyanka Khati, Deep Chandra Suyal, Kahkashan Perveen, Faheema Khan, Jayanthi Barasarathi

**Affiliations:** ^1^Department of Biotechnology, Keral Verma Subharti College of Science, Swami Vivekanand Subharti University, Meerut, India; ^2^Department of Microbiology, CBSH, GBPUAT, Pantnagar, Uttarakhand, India; ^3^Agricultural Microbiology Laboratory, Crop Production Division, ICAR VPKAS, Almora, Uttarakhand, India; ^4^Department of Science and Humanities, Vidyadayini Institute of Science, Management & Technology, Bhopal, Madhya Pradesh, India; ^5^Department of Botany and Microbiology, College of Science, King Saud University, Riyadh, Saudi Arabia; ^6^Faculty of Health and Life Sciences, INTI International University, Nilai, Negeri Sembilan, Malaysia

**Keywords:** chickpea, filed trial, phosphate solubilizers, RT-PCR, principal component analysis, Agricultural practices, Sustainable land management

## Abstract

The phosphate solubilizing properties of *Lysinibacillus macroides* ST-30, *Pseudomonas pelleroniana* N-26, and *Bacillus cereus* ST-6 were tested for the chickpea crop of the Tarai region of Uttarakhand. These microbially inoculated plants have shown significant (*p* > 0.05) improvement in the plant health and crop health parameters, *viz.*, root length, shoot length, fresh weight, dry weight, nodule number, nodule fresh weight, nodule dry weight, chlorophyll content, and nitrate reductase. The highest shoot length (46.10 cm) and chlorophyll content (0.57 mg g^−1^ fresh weight) were observed in ST-30 at 75 DAS with 20 kg P_2_O_5_/ha. Similarly, for plant P content, an increase of 90.12% over control was recorded in the same treatment. Treatments consisting of *Lysinibacillus macroides* ST-30 along with 20 kg/ha P_2_O_5_ were found to be most suitable as phosphatic fertilizer. Conclusively, sustainable agriculture practices in the Tarai as well as the field region may be developed based on a strategy of exploring microbial inoculants from the pristine region of the Western Himalayas. The presence and abundance of bacterial inoculants were confirmed through qRT-PCT. We conclude that the effective plant growth-promoting bacterium *Lysinibacillus macroides* ST-30 broadens the spectrum of phosphate solubilizers available for field applications and might be used together with 20 Kg/ha P_2_O_5_.

## 1 Introduction

Chickpea (*Cicer arietinum*) is a significant staple crop, providing a valuable source of protein and essential nutrients for millions of people worldwide. Chickpea contains 18–22% protein, 52–70% carbohydrate, 4–10% fat, and a sufficient quantity of minerals, calcium, phosphorus, iron, and vitamins. In addition, it is also important for sustainable agriculture as it improves the physico-chemical and biological properties of the soil. It improvises the soil structure due to deep roots and fixes approximately 25–30 kg N ha^−1^ through symbiosis (Reddy and Reddy, 2005), minimizing dependency on chemical fertilizers. A sufficient supply of phosphorus to the plant increases the nodulation and fastens its maturity. Phosphorus as a micronutrient also helps in root and shoot development and improves photosynthesis and other physico-biochemical processes (Singh et al., 2018).

However, a substantial proportion of soil phosphorus remains inaccessible to plants due to its insoluble form. Phosphate-solubilizing bacteria (PSB) possess the unique ability to convert insoluble phosphorus into a soluble form, thus enhancing its bioavailability to plants. The most significant phosphate-solubilizing bacterial genera are reported, such as *Azotobacter, Bacillus, Beijerinckia, Burkholderia, Enterobacter, Erwinia, Flavobacterium, Pseudomonas, Microbacterium, Serratia*, *Rhizobium, Bradyrhizobium, Salmonella, Alcaligenes, Chromobacterium, Arthrobacter, Streptomyces, Thiobacillus,* and *Escherichia* (Zhu et al., 2011). As per acid production theory, phosphate solubilization by phosphate-solubilizing microorganisms (PSMs) takes place through the synthesis of various organic acids such as malic, succinic, glyoxalic, fumaric, α-keto butyric, tartaric, citric, oxalic, 2-keto gluconic, and gluconic acids. The release of organic acid acidifies the medium (Puente et al., 2004; Singh et al., 2012; Rani et al., 2013). Different microorganisms produce different types of organic acid. The organic acid that is released in culture filtrate reacts with the insoluble phosphate (Ahmed and Kibret, 2014).

Gluconic acid is the major acid secreted by P solubilizers and synthesized by a mechanism involving direct oxidation of glucose through two key proteins, namely membrane-bound quinoprotein and glucose dehydrogenase (GDH) (Kim et al., 1997; Patel et al., 2008). GDH requires pyrroloquinoline quinone (PQQ) as a cofactor, which is the product of a *pqq* operon comprised of six genes (*pqq*A, B, C, D, E, and F) in *Klebsiella pneumonia, Enterobacter intermedium* 60-2G, *and Rahnella aquatilis* (Kim et al., 1998, 2003). PQQ is essential for the formation of holoenzymes, which lead to the production of gluconic acid from glucose. Han et al. (2008) have revealed a lack of 2-keto gluconic acid production through GA oxidation by the enzyme gluconic acid dehydrogenase due to the inactivation of *pqq* genes in *Enterobacter intermedium* 60-2G. Polymerase chain reaction (PCR) studies were conducted in the *S. marcescens* CTM 50650 strain (Farhat et al., 2009) to check the presence of genes involved in the expression of mucopolyssacharides (MPS) via the activation of the direct oxidation pathway of glucose (GDH encoded by the *gdh* gene and pyrroloquinoline quinone *(pqq)* genes involved in the biosynthesis of the required PQQ cofactor). This capacity makes PSB a promising candidate for improving nutrient uptake efficiency and subsequently influencing the overall productivity of crops such as chickpeas. Looking into the importance of legume crops for soil health management and their requirement for phosphate, an experiment was designed to optimize the level of P fertilizers along with PSB. This research aims to provide insight into the impact of P solubilizers on the health and nodulation efficiency of chickpeas, which are responsible for nitrogen fixation by the plant. The nitrogen-fixing potential was also measured through the nitrate reductase test of the plant. The dominance of the inoculated bacterium was also confirmed throughout the experiment through quantitative reverse transcriptase-polymerase chain reaction (qRT-PCR).

## 2 Materials and methods

### 2.1 Strain and culture condition

Three strains, namely ST-30 (*Lysinibacillus macrolides;* accession No. KX396054), ST-06 (*Bacillus cereus;* accession No MF496242), and N-26 (*P. pelleroniana*; accession No. JN055435), were used for the field study. Phosphate solubilizer and diazotrophic isolate, strain N-26, originally isolated from the soil of the Nainital region (2,084 m, 29.38°N 79.45°E) of Uttarakhand, was obtained from departmental culture collection and used in this study (Tomer et al., 2017).

### 2.2 Plant growth promotion studies under field conditions on chickpea (*Cicer arietinum*, PG-186)

The field trial was performed in Pantnagar (altitude of 343.84 m above mean sea level, 29^0^N latitude and 79.3°E longitudes), which falls under the foothills of “Shivalik” ranges of “Himalaya” a narrow belt called “Tarai.” Field Trial The experiments on chickpea (*Cicer arietinum*, PG-186) were conducted between November and April (2015–2016) using a randomized block design (RBD) at the Norman E. Borlogue Crop Research Centre of G.B. Pant University of Agriculture and Technology, Pantnagar, Dist. Udham Singh Nagar (Uttarakhand). The growth promotory efficiency and P solubilization potential of these selected bacterial strains were checked under natural field conditions. Seeds of chickpea (PG-186) were surface sterilized prior to sowing (Goel et al., 2002). The seeds were sown on 26 November 2015, and the crop was harvested on 13 April 2016. There were a total of 12 treatments (T1–T12) (Table 1).

**Table 1:** Treatment detail in field trial.

No	Treatment	Details
1	T1	Uninoculated control (without PSB and P_2_O_5_)
2	T2	Uninoculated +20 kg ha^−1^ of P_2_O_5_
3	T3	Uninoculated +40 kg P_2_O_5_ ha ^−1^
4	T4	PSB ST-30 + 0 P_2_O_5_
5	T5	PSB ST-30 + 20 kg ha^−1^ of P_2_O
6	T6	PSB ST-30 + 40 kg ha^−1^ of P_2_O
7	T7	PSB N-26 + 0 P_2_O_5_
8	T8	PSB N-26 + 20 kg ha^−1^ P_2_O_5_
9	T9	PSB N-26 + 40 kg ha^−1^ P_2_O_5_
10	T10	PSB ST-06 + 0 P_2_O_5_
11	T11	PSB ST-06 + 20 kg ha^−1^ P_2_O_5_
12	T12	PSB ST-06 + 40 kg ha^−1^ P_2_O_5_

Thirty-six plots of 8.4 m^2^ were made. All analyses were carried out in triplicate. After sowing, agronomical plant parameters (root length, shoot length, fresh weight, dry weight, nodule number, nodule fresh weight, and nodule dry weight), leaf nitrate reductase activity (Hageman and Hucklesby, 1971), total leaf chlorophyll content (Hiscox and Israelstam, 1979), and total plant P content (Jackson, 1962) were checked at 40, 60, and 75 DAS. Shoot length and root length were measured by fully expanding the leaves and roots. The fresh weight of the plant, nodule number, and nodule fresh weight were weighed immediately after harvesting, while dry weight was measured after oven drying the samples at 50°C for 10 days to get a constant weight. Simultaneously, rhizospheric soil samples were collected for bacterial community analysis by sterile spatula in sterile polythene bags and transported to the laboratory aseptically in cold conditions. Each soil sample was collected in triplicates, which were later mixed to make a single bacterial composition per treatment.

### 2.3 Statistical analysis

All the data from the field trial study was statistically analyzed using the statistical program analysis of variance (ANOVA) and general linear model procedure (SPSS, ver. 16.0) to reveal the significant effect of different treatments. Duncan’s multiple range test (DMRT) was applied to determine the difference between individual events at a significant difference (*p* ≤ 0.05). Principal component analysis was performed with the help of JMP 9 software.

### 2.4 Bacterial community analysis through real-time PCR (q PCR) analysis

The effect of PSBs on native microflora was assessed using qRT-PCR. The rhizospheric soil sample (≤ 15 cm) was collected from the rhizosphere of chickpea from each plot and pooled for DNA isolation, as previously described in the article.

The copy number of 16S rDNA from the collected soil samples was quantified using the primer set 16S F/R (5′-CCTACGGGAGGCAGCAG-3′ and 5’-ATTACCGCGGCTGCTGG-3′) with the iCycler iQ™ Multicolor (Bio-Rad Lab, Hercules, CA, USA) instrument, employing SYBR green chemistry (Kumar et al., 2014).

## 3 Results

### 3.1 Impact of P solubilizers on shoot and root growth

**Figure 1:**
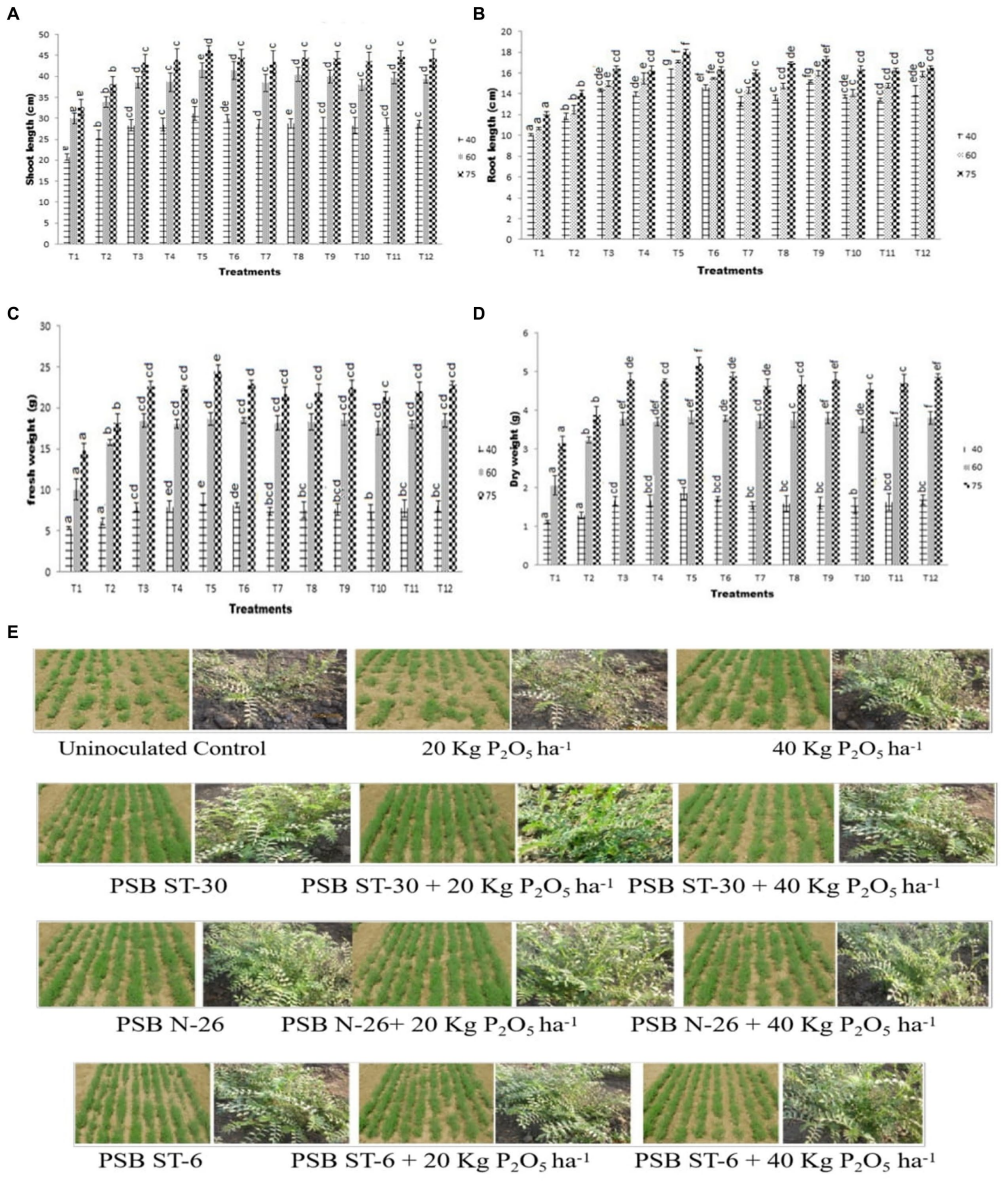
Effect of PSB (ST-30, N-26, and ST-6) on **(A)** shoot length (cm) and **(B)** root length (cm) at 40, 60, and 75 DAS, respectively, **(C)** effect of PSB (ST-30, N-26, and ST-6) on (A) fresh weight (g) and **(D)** dry weight at 40, 60, and 75 DAS, respectively, and **(E)** comparative effect of PSB ST-30, N-26, and ST-6 on plant health of *Cicer arietinum* L. var. PG-186 at 40 DAS.

**Figure 2:**
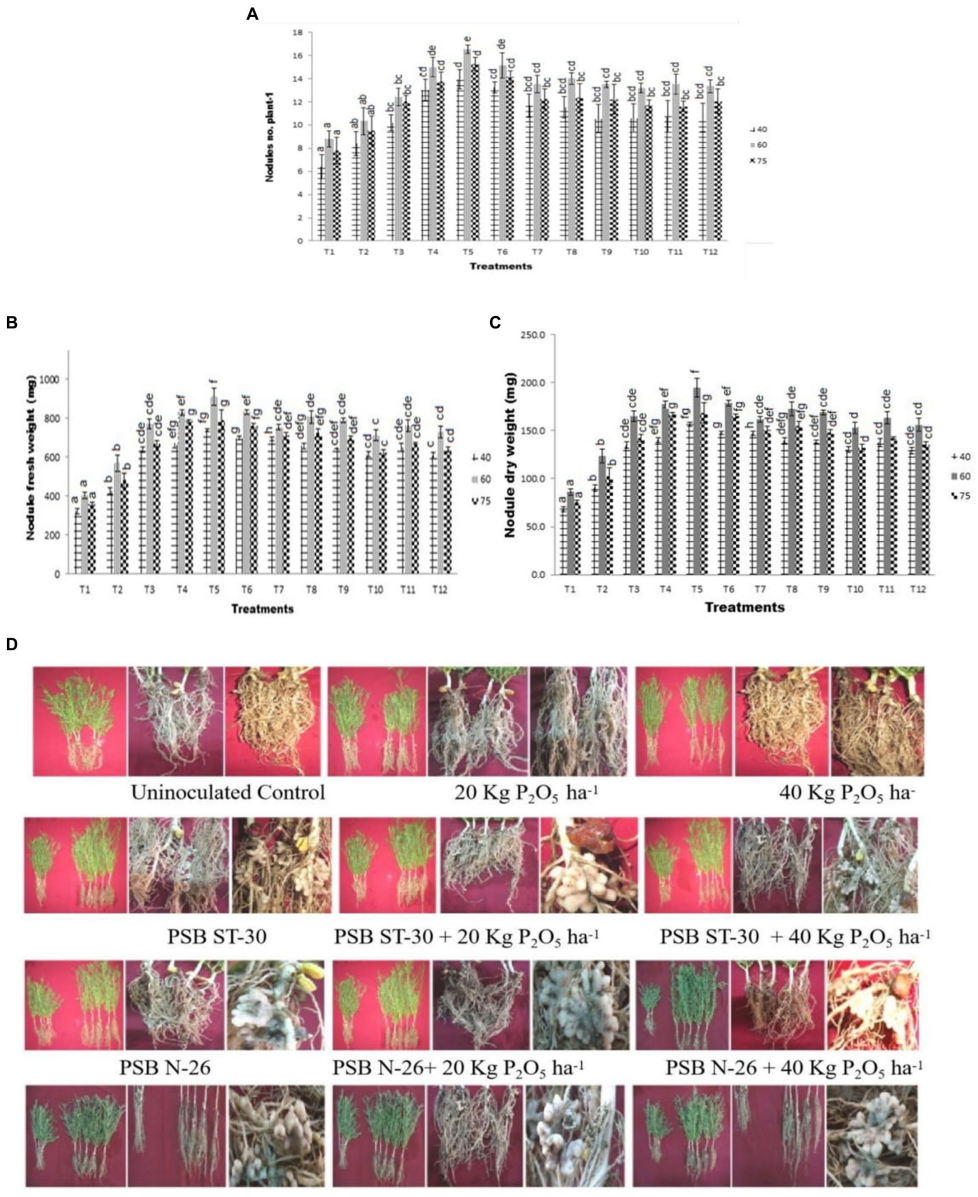
Effect of PSB (ST-30, N-26, and ST-6) on **(A)** nodule count plant^−1^,’ **(B)** nodule fresh weight (g), and **(C)** nodule dry weight (g) at 40, 60, and 75 DAS, respectively, **(D)** comparative effect of PSB *viz.* ST-30, N-26, and ST-6 on shoot length, root length, and nodulation of *Cicer arietinum* L. var. PG-186 under different treatments at 60 DAS.

A significant increase in shoot length was observed in bio-inoculant-treated plants over the control. The highest shoot length was observed in ST-30 (46.10 cm) at 75 DAS with 20 Kg P_2_O_5_/ha which was even greater than ST-30 with 20 Kg P_2_O_5_/ha and almost similar to ST-30. Similarly, in the case of P-06 and N-26 treatments, no significant effect of phosphate fertilizer was observed (Figures 1A,C, 2).

Similarly, maximum root length was observed in ST-30 along with 20 Kg P_2_O_5_/ha at different sampling stages, which was approximately 50% higher than the control. The treatment of S-06 and N-26 almost nullified the need for P fertilizers in terms of root length, as similar results were obtained in treatments that were inoculated without any chemical fertilizers and another treatment which was inoculated along with 20 and 40 kg ha^−1^ of chemical fertilizers (Figures 1B–D, 2).

3.2. Fresh weight and dry weight

A significant increase in fresh weight was observed in bio-inoculant-treated plants over their control. The highest fresh weight of 8.83 g was recorded in a treatment of ST-30 with 20 Kg P_2_O_5_/ha. The highest recorded fresh weight growth is 65.73% in terms of percentage increase over control at 40 DAS (Figure 1C). A similar pattern was observed in 60 and 75 DAS, respectively. The pattern of fresh weight was followed in dry weight, where maximum dry weight was again obtained in ST-30 with 20 Kg P_2_O_5_/ha at 40 (1.85 g), 60(3.81 g), and 75 (5.21 g) DAS (Figure 1D).

### 3.2 Effect of P solubilizers on nodules of chickpea

Maximum numbers of nodules were observed in ST-30 along with 20 Kg P_2_O_5_/ha at 60DAS which decreased at 75DAS which shows the degeneration of nodules after 60 days. The increase in nodulation was about more than 100% with respect to control. A significant increase in nodulation was observed after the inoculation of different PSBs. This shows the role of phosphate metabolism in the nodulation efficiency of legumes. A similar trend was also observed in nodule fresh weight and dry weight. A significant decline in nodule count, fresh weight, and dry weight was recorded at 75 DAS, showing degeneration of nodules after a certain time. The results also correlated with shoot length and root length (Figures 2A–C). The three bacteria, individually and in consortium, showed more vigor in terms of nodulation and nitrogen content than non-inoculated plants (Figure 2D).

### 3.3 Leaf chlorophyll content and nitrate reductase activity

**Figure 3:**
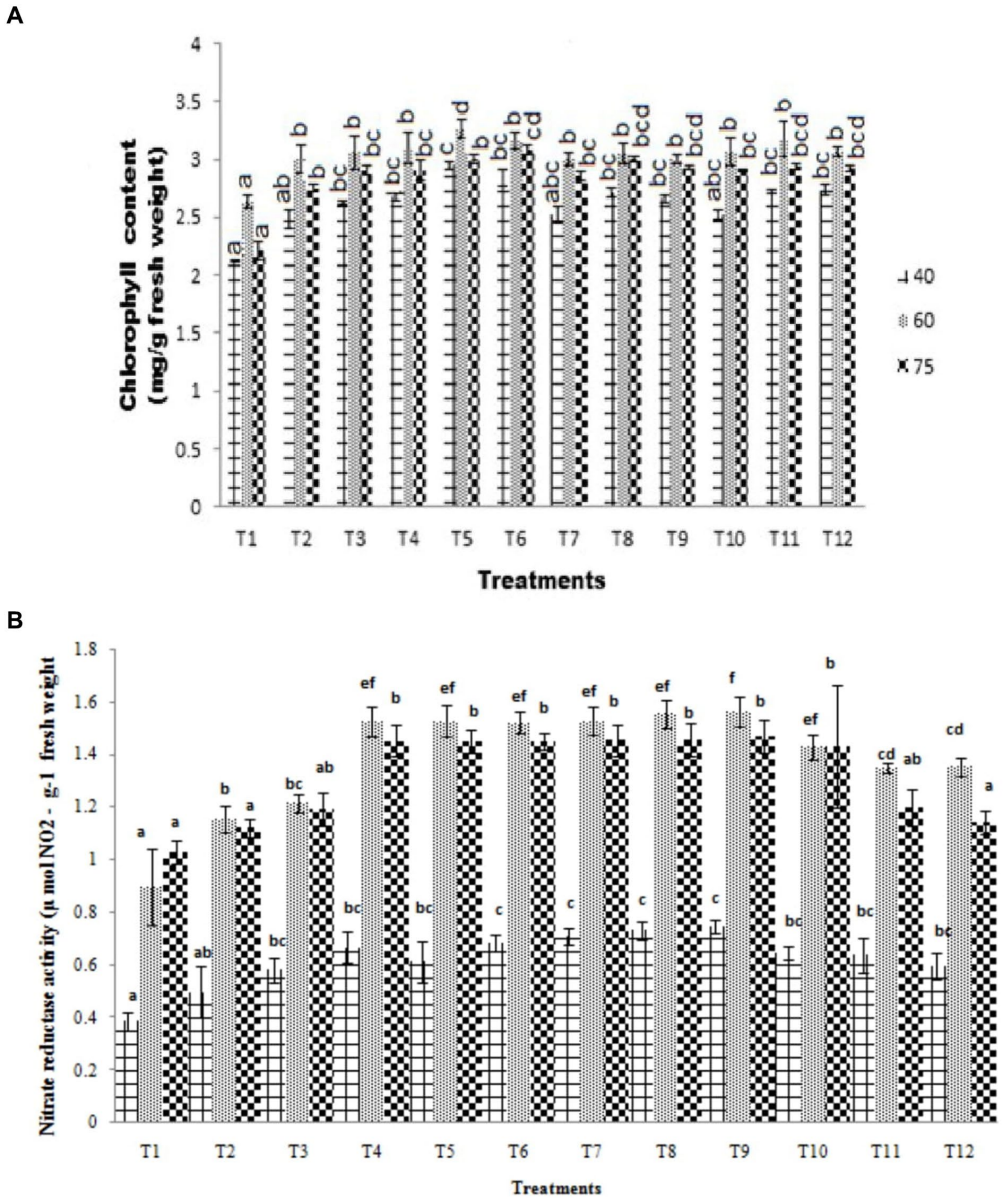
**(A)** Effect of PSB (ST-30, N-26, and ST-6) on the chlorophyll content (mg g^−1^ fresh weight) at 40, 60, and 75 DAS, respectively. **(B)** Effect of PSB (ST-30, N-26, and ST-6) on nitrate reductase activity (μ mol NO₂ g^−1^ fresh weight) at 40, 60, and 75 DAS, respectively.

Plant chlorophyll and nitrate reductase activity were studied to show plant growth promotion by bio-inoculants. At 40 DAS, the chlorophyll content of absolute control was measured as 2.12 mg g^−1^ fresh weight, while the highest chlorophyll content of 2.57 mg g^−1^ fresh weight was recorded in ST-30 along with 20 Kg P_2_O_5_/ha. This is 38.76% in terms of percentage increase over control. This is followed by treatment having ST-30 with 40 Kg P_2_O_5_/ha (33.55% increase over control), which was at par with ST-06 with 40 Kg P_2_O_5_/ha (28.77% increase over control). Chlorophyll content increased when chemical fertilizer over the bio-inoculum was provided up to 20 kgha^−1^ but did not show a significant increase and even sometimes decreased at 40 kgha^−1^ (Figure 3). A decrease in chlorophyll content was also recorded at 75 DAS, which shows the maturation phase of the crop.

Nitrate reductase, the first in a series of enzymes that reduce nitrate to ammonia, is sensitive to a number of environmental factors (McAllister et al., 2012). Activity varies under the light intensity influence, carbon dioxide levels, temperature variation, H_2_O availability, and nitrate availability (Fageria and Baligar, 2005). Croy and Hageman (1970) took it as a biochemical marker for prediction yield and protein concentration, which was later followed by Zhang et al. (2015) and others. Further nitrogen (N) uptake by crops can be controlled by their growth rate and can vary according to the requirement of nitrogen incorporation in plants at a particular growth period (Barret et al., 2000). Maximum activity was recorded in PSB N-26 + 40 kg P_2_O_5_ ha-1 (0.75 μ mol NO_2_ g-1 fresh weight) at all the sampling intervals, followed by PSB N-26 + 20 kg P_2_O_5_ ha ^−1^ (0.73 μ mol NO_2_ g^−1^ fresh weight) at 40 DAS, which was 1.56 and 1.55 at 60DAS and 1.47 and 1.46 at 75DAS, respectively, for PSB N-26 + 40 kg P_2_O_5_ ha ^−1^ and PSB N-26 + 20 kg P_2_O_5_ ha ^−1^, which was more than 100% increase w.r.t control (Figure 3).

Nitrate reductase assay was performed to study the assimilation of nitrate in plants, whereas chlorophyll content was measured to monitor plant health. Plant nitrate reductase activity was found to have a positive correlation with soil nitrate concentration. Leaf nitrate reductase and chlorophyll content both increased in the plants treated with bio-inoculums (ST-30, N-26, and ST-6) when compared to uninoculated absolute control and treatments containing only chemical fertilizers. The temporal pattern of increase in leaf nitrate reductase and chlorophyll content in treatment plants at different intervals was 40 < 60 > 75 (DAS) in all crops with maximum nitrate reductase and chlorophyll content at 60 DAS. Control plants also followed the same trend.

### 3.4 Principal component analysis

**Figure 4:**
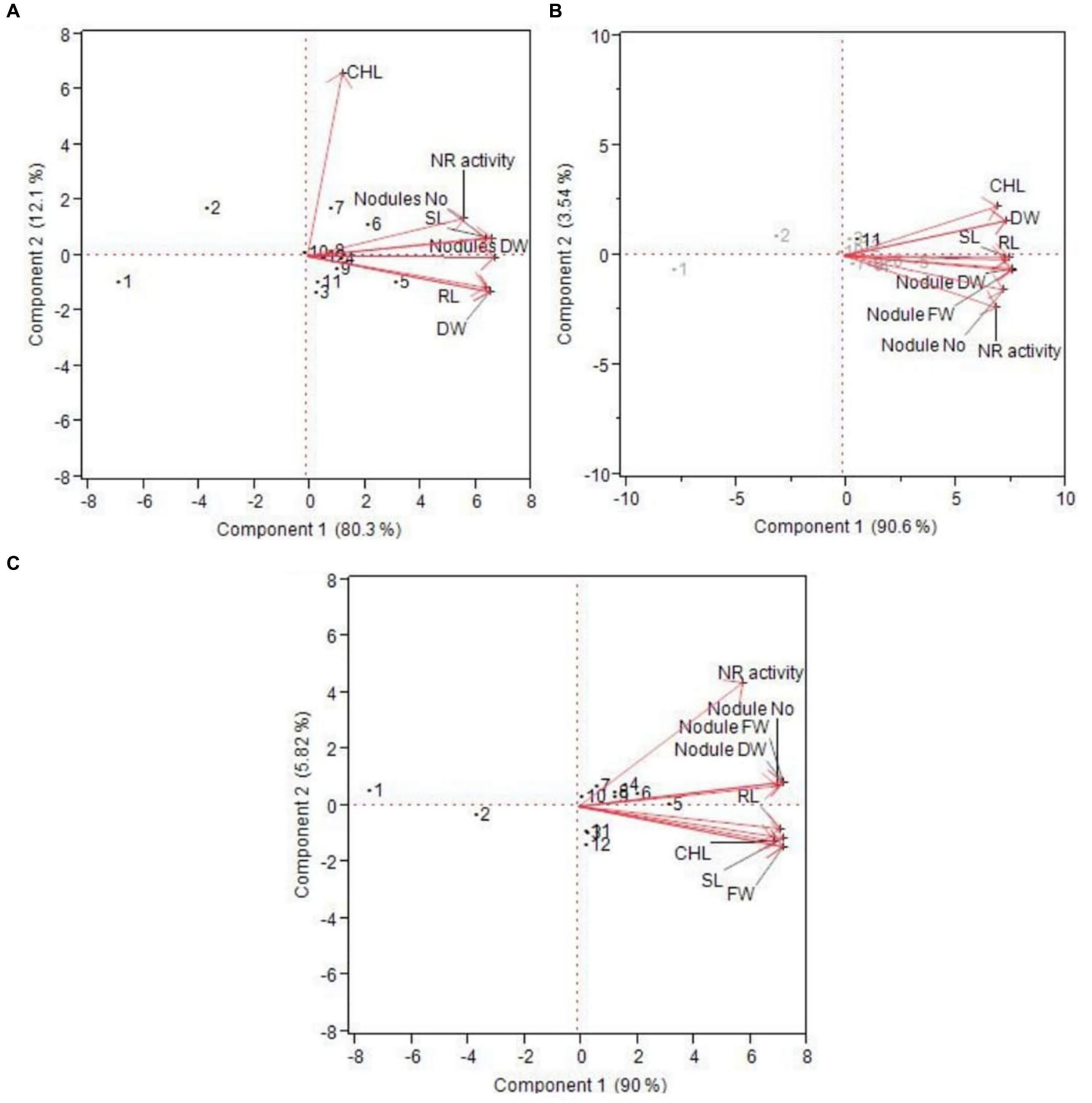
Principal component analysis of different plant health parameters at **(A)** 40 days, **(B)** 60 days, and **(C)** 75 days of treatments.

The PCA analysis shows a positive correlation of all the treatments with different parameters (chlorophyll content, nitrate reductase activity, fresh weight and dry weight of the plant, nodule fresh and dry weight, nodule number, and shoot and root length) except T1 [uninoculated control (without PSB and P_2_O_5_)] and T2 (uninoculated +20 kg ha^−1^ of P_2_O_5_), which did not show any correlation with treatments (Figure 4). This trend was observed in different time intervals, i.e., 40 days, 60 days, and 75 days. All the other treatments had a positive correlation with different parameters, showing the positive impact of all other treatments on different parameters. Similarly, Rhodes et al. (2014) also evaluated green manure crops for the management of nematodes in soil through PCA analysis.

### 3.5 Plant P content

**Figure 5:**
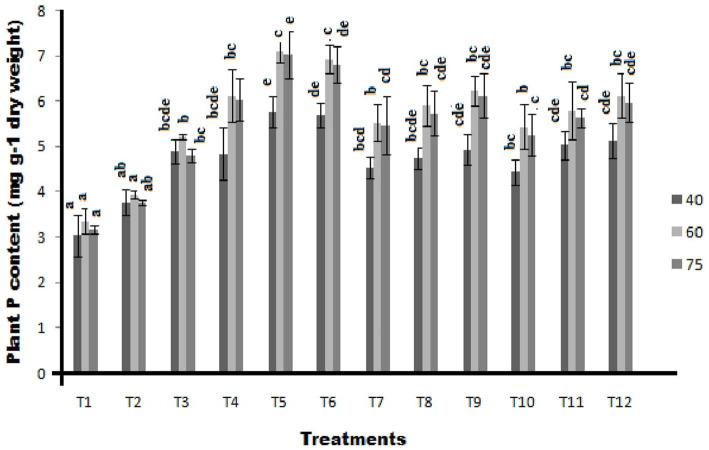
Effect of PSB (ST-30, N-26, and ST-6) on plant P content (mg g^−1^ dry weight) at 40, 60, and 75 DAS, respectively.

Significantly, the highest plant P content of 5.77 mg g^−1^ dry weight was recorded in ST-30 along with 20 Kg P_2_O_5_/ha which was a 90.12% increase over control. This is followed by treatment having ST-30 and 40 Kg P_2_O_5_/ha with 87.92% (5.70 mg g^−1^ dry weight) increase over absolute control, which is at par with treatment having ST-06 with 40 Kg P_2_O_5_/ha with 69.24% increase over control at 40DAS (Figure 5). Treatments containing N-26 with a blend of 20 Kg P_2_O_5_/ha and 40 Kg P_2_O_5_/ha have shown a 56.05 and 62.65% increase over control, respectively. Similarly, treatments consisting of ST-06 with the blend of 20 Kg P_2_O_5_/ha showed a 65.94% increase over control.

A similar trend was observed at 60 DAS, where the highest plant P content of 7.10 mg g^−1^ dry weight was recorded in ST-30 along with 20 Kg P_2_O_5_/ha. This is 111.73% in terms of percentage increase over control, followed by treatment having ST-30 and 40 Kg P_2_O_5_/ha with 106.76% increase over absolute control, which is at par with treatment having N-26 with 40 Kg P_2_O_5_/ha with 85.89% increase over control with recorded plant P content 6.23 mg g^−1^ dry weight. The trend was followed even at 75 DAS.

### 3.6 Monitoring of bacterial community for the diversity and occurrence of inoculated strain

**Figure 6:**
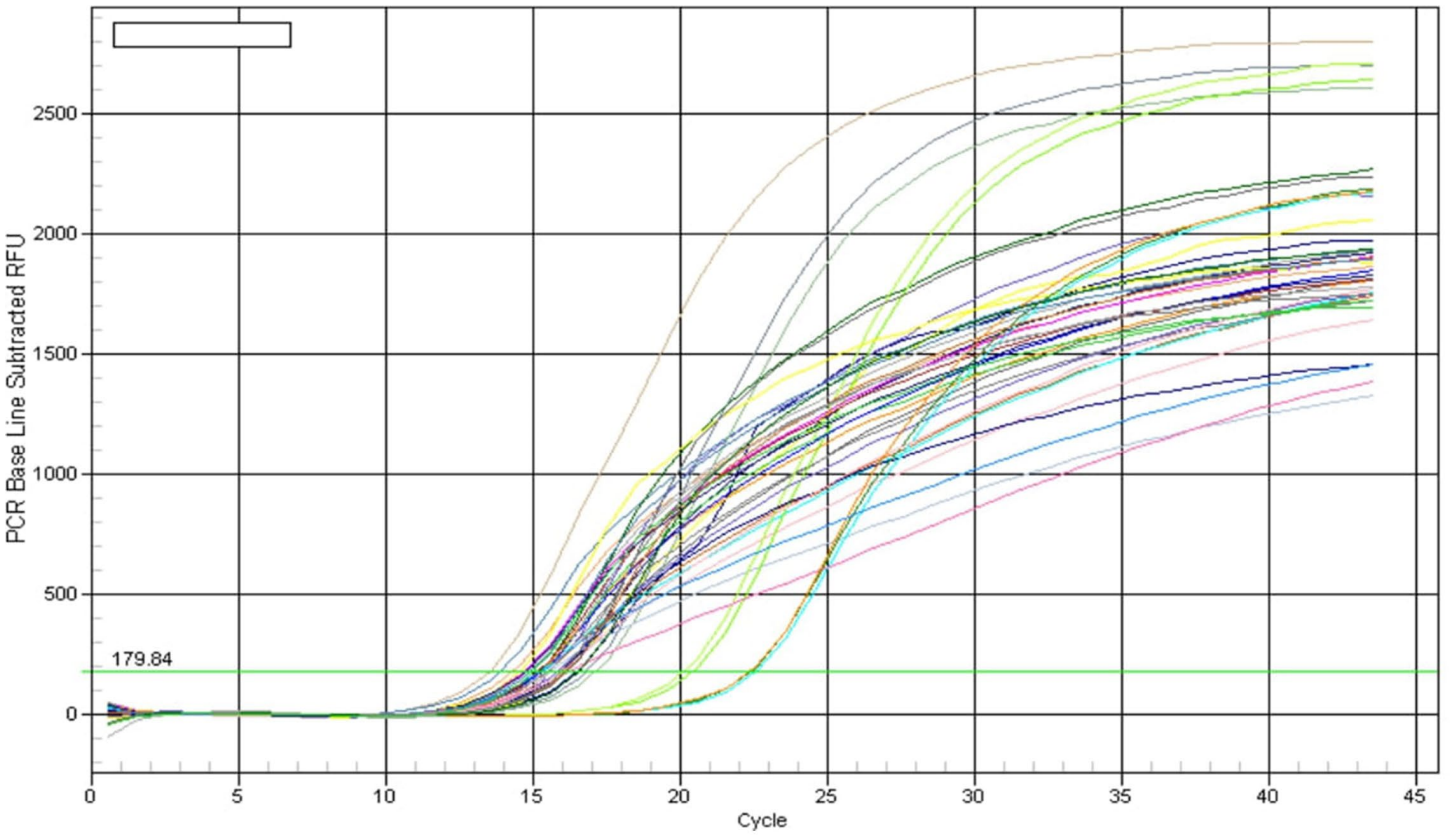
Amplification curve of real-time PCR of soil samples collected at 0, 40, 60, and 75 days after sowing, respectively.

Quantification of soil samples showed that soil treated with ST-30 had a maximum of 16 S rRNA gene copy no. at every stage of sampling. Treatment having ST-30 as PSB has 6.26 × 10 (Croy and Hageman, 1970), 9.41 × 10^9^, and 4.77 × 10^10^ at 40, 60, and 75DAS, respectively (Table 2). The results of total bacterial abundance in soil at different times are shown in Figure 6. Quantification showed a linear relation (*R*^2^ = 0.993) between the log value of bacterial genomic DNA and real-time PCR threshold cycles over the range of examined DNA concentrations. Soil samples of treatment consisting of 20 Kg P_2_O_5_ ha^−1^ and 40 Kg P_2_O_5_ ha^−1^ had the lowest 16 S ribosomal gene copy no. at all stages of sampling. Furthermore, all PSB-treated treatments had remarkably higher 16 S rRNA gene copy numbers than uninoculated control and only chemical fertilizer-treated treatments.

**Table 2:** Quantification of the 16 S r RNA gene from different treatment soils at different time intervals using real-time PCR.

	Copy no. of ribosomal genes at		
	40 DAS^a^	60 DAS^a^	75DAS^a^
Presowing soil	2.21 × 10^9^		
Uninoculated control	3.41 × 10^9^	3.90 × 10^9^	5.51× 10^9^
20Kg P_2_O_5_ ha^−1^	2.20 × 10^8^	7.10 × 10^8^	4.25 × 10^9^
40Kg P_2_O_5_ ha^−1^	1.21 × 10^8^	7.41 × 10^8^	9.51× 10^8^
ST-30	6.26 × 10^9^	9.41 × 10^9^	4.77× 10^10^
ST-30 + 20Kg P_2_O_5_ ha^−1^	4.10 × 10^9^	8.27 × 10^9^	2.78× 10^10^
ST-30 + 40Kg P_2_O_5_ ha^−1^	9.71× 10^8^	3.53× 10^9^	1.58 × 10^10^
N-26	4.20× 10^9^	7.59 × 10^9^	2.51× 10^10^
N-26 + 20Kg P_2_O_5_ ha^−1^	4.21 × 10^9^	7.417× 10^9^	2.54× 10^10^
N-26 + 40Kg P_2_O_5_ ha^−1^	3.24 × 10^9^	4.48 × 10^9^	9.54× 10^9^
ST-6	5.91 × 10^9^	6.40 × 10^9^	9.51× 10^9^
ST-6 + 20Kg P_2_O_5_ ha^−1^	3.45 × 10^9^	5.45 × 10^9^	8.54× 10^9^
ST-6 + 40Kg P_2_O_5_ ha^−1^	9.67 × 10^8^	2.40 × 10^9^	7.54× 10^9^

^a^The formula used for copy no. calculation is: number of copies = (amount ×N/length ×1×10^9×^m).

Where *m* = average weight of a base pair (bp) is 650 Daltons, *N* = Avogadro’s number (6.022 × 10^23^ molecules/mole).

## 4 Discussion

The results show the positive implications of microbial inoculants on plant health, which is ultimately related to its vigor and productivity. A similar study was also conducted by Rajwar et al. (2018) using psychrotolerant *Pseudomonas jesenii* MP1 and *Acinetobacter* sp. ST02 against chickpeas. They obtained promising results in terms of plants’ agronomical and biochemical properties. The positive applications of PGPR on plant fresh and dry weights are multifaceted, encompassing improved nutrient uptake, induced systemic resistance, auxin production, enhanced water use efficiency, stress tolerance, and increased photosynthetic efficiency. The effect of PGPR on growth, nodulation, and nutrient accumulation in lentils was also studied by Zafar et al. (2012). They proposed the application of PGPR for the growth and nutrient accumulation of crops as an alternative to chemical fertilizers. The nodulation efficiency was improved after the application of P solubilizers, as pulses are observed to be heavy P feeders, which are required for nodulation and hence the growth of pulses. Flores-Duarte et al. (2022) also studied the impact of nodulation-enhancing rhizobacteria on *Medicago sativa.* They studied the impact of three strains, *viz., Pseudomonas* sp. L1, *Chryseobacterium soli* L2, and *Priestia megaterium* L3, on *Medicago sativa* under greenhouse conditions.

The highest chlorophyll content was observed when chemical fertilizer (up to 20 kgha^−1^) over the bio-inoculum was provided but did not show a significant increase at 40 kgha^−1^. Chlorophyll content is a measure of plant health as it is involved in photosynthesis (Shukla et al., 2015). Chlorophyll content in leaves also relies on phosphorous concentration, as it helps the plant survive under adverse conditions. Furthermore, its deficiency reduces plant protein and chlorophyll content. Phosphorous scarcity is also responsible for alternations in chlorophyll components, thus affecting absorption. However, the biochemical characteristics and biosynthesis of chlorophyll depend on the uptake of optimal phosphorous (Razaq et al., 2017). A noteworthy upsurge in the chlorophyll content was noticed in lettuces planted in soil introduced with superphosphate (Azzi et al., 2017). Similarly, NR activity was highest in 60 days, along with bacterial inoculation. These results revealed that the inoculation of bio-inoculums (ST-30, N-26, and ST-6) in soil facilitates plant nutrient uptake from the soil, which leads to better plant growth. Recently, Azzi et al. (2017) reported that phosphate fertilizer application in alkaline soil contaminated by cadmium boosted the NR activity in *Lactuca sativa*. Furthermore, diazotrophs are capable of altering nitrate and ammonium in plants, resulting in the induction of enzymes required for nitrogen metabolism (Dos Santos et al., 2017). The highest activity in the case of treatments having N-26 can be the reason for it, as it is a diazotroph too, besides PSB (Agarwal, 2013). Nevertheless, NR activity can be used as a parameter for nitrogen use efficiency in the selection of genotypes responsive to nitrogen fertilization or adapted to nitrate restriction (Sinha et al., 2015). Chen et al. (2016) revealed that phosphate-solubilizing bacteria help enrich the soil microbial communities by accelerating litter decay and nitrate release, thus increasing the available nitrate content in the soil, which is directly proportional to plant nitrate reductase activity. Furthermore, NR activity can be used as a biochemical marker for predicting grain yield and grain protein production (Boulter, 2012; Krishnamoorthy et al., 2013).

The application of phosphorus seemed to have a direct effect on the P content of plant samples. In all plants, plant P content was higher than the uninoculated control. Bargaz et al. (2021) also studied the impact of PSB on the below group performance of crops for their health and improved P acquisition from the soil. Stephan et al. (2015) also proved that PSB increases the soil phosphorus content, making it available for plant uptake. In all bioagents (ST-30, N-26, and ST-6) treated crops, plant P content was increasing with time up to 60 DAS but there after at 75 DAS it showed a slight declination. It revealed that plants have the highest ‘P’ demand in the young stage, at the time of flowering and nodule formation (Divito and Sadras, 2014). In a similar study, Bechtaoui et al. (2020) studied the impact of PGPR co-inoculation on bean production under varying phosphorous availability. They observed that regardless of the applied phosphorus source, co-inoculation of PGPr significantly increased biomass and phosphorus concentrations in plants as well as in pods. In another study by Zineb et al. (2019), inoculation of PSB was observed to improve the uptake and effectiveness of rock phosphate as a fertilizer.

The qPCR analysis also confirmed the dominance of microbial inoculants up to 60DAS, which is a good indicator of their activity in the field. The impact of bio-inoculant on soil microbial communities has been studied earlier using DGGE (Herrmann et al., 2012), qPCR (Babic et al., 2008), ribosomal intergenic spacer analysis “RISA” (Schumpp and Deakin, 2010), and other techniques.

## 5 Conclusion

Microorganisms play a pivotal role in the functioning of crops by influencing their physiology and development. Rhizosphere microorganisms promote plant growth and protect plants from pathogen attack by different mechanisms. In this perspective, an exploration of cold-adaptive bacteria as representative candidates for plant growth promotion and nutrient acquisition was conducted and could be a better alternative for improved crop sustainability. The inoculated bacterial strains proved their potential as a better alternative to chemical phosphatic fertilizers in terms of plant growth, nodulation efficiency, and plant nutrient uptake. The plant biochemical parameters (chlorophyll and nitrate reductase tests) also correlate with agronomic data, which shows that bio-inoculants not only help growth promotion but also have properties that improve stress tolerance in host plants. The dominance of inoculated P solubilizers throughout the experiment shows they have the potential to fight the competition with native microflora and have a good shelf life in field conditions, advocating their application as commercial fertilizer for the future.

## Data Availability

The original contributions presented in the study are included in the article/Supplementary material, further inquiries can be directed to the corresponding author.
