# Supplementary material for: Relative multi-beneficial effect of MOs on plant health of chickpea (Cicer arietinum L. var. PG-186)
Source: Front Microbiol. 2024 Aug 27;15:1452553. doi: 10.3389/fmicb.2024.1452553 (PMC11385864; doi:10.3389/fmicb.2024.1452553)
Supplement: Supplementary file 1 [file Data_Sheet_1.docx]

**Supplementary table 1: Effect of PSB (ST-30, N-26, ST-6) on shoot length (cm) of *Cicer arietinum* L. var. PG-186**

| **Treatment** | **40 DAS** | **60 DAS** | **75DAS** |
| --- | --- | --- | --- |
| Uninoculated control | 20.80± 0.75**^a^** | 29.97± 1.24**^a^** | 32.63± 1.86**^a^** |
| 20 kg P_2_O_5_ ha ^-1^ | 26.07± 0.99**^b^** (25.32) | 33.87± 1.24**^ab^** (13.00) | 38.07± 1.87**^ab^** (16.66) |
| 40 kg P_2_O_5_ ha ^-1^ | 28.33± 1.37**^bc^** (36.22) | 38.53± 1.33**^bc^** (28.57) | 43.23± 2.03**^bc^** (32.50) |
| PSB ST-30 | 28.43± 1.54**^bc^** (36.70) | 38.60± 2.18**^bc^** (28.80) | 44.03± 2.57**^bc^** (34.95) |
| PSB ST-30 + 20 kg P_2_O_5_ ha ^-1^ | 31.13± 1.56**^c^** (49.68) | 41.50± 1.64**^c^** (38.47) | 46.10± 1.11**^c^** (41.28) |
| PSB ST-30 + 40 kg P_2_O_5_ ha ^-1^ | 30.00± 0.93**^bc^** (44.23) | 41.33± 2.12**^c^**  (37.92) | 44.47± 1.92**^bc^** (36.28) |
| PSB N-26 | 28.63± 1.02**^bc^** (37.66) | 38.43± 2.09**^bc^** (28.24) | 43.47± 2.58**^bc^** (33.21) |
| PSB N-26 + 20 kg P_2_O_5_ ha ^-1^ | 28.77± 1.16**^bc^**  (38.30) | 40.43± 1.73**^c^** (34.91) | 44.40± 1.75**^bc^** (36.07) |
| PSB N-26 + 40 kg P_2_O_5_ ha ^-1^ | 28.90± 1.25**^bc^** (38.94) | 39.97± 1.54**^c^**  (26.68) | 44.33± 1.57**^bc^** (35.87) |
| PSB ST-6 | 28.30± 1.90**^bc^**  (36.06) | 37.97± 1.27**^bc^** (26.68) | 43.60± 2.07**^bc^** (33.62) |
| PSB ST-6+ 20 kg P_2_O_5_ ha ^-1^ | 28.50± 1.50**^bc^** (37.02) | 39.67± 1.35**^c^** (32.35) | 44.57± 1.59**^bc^** (36.58) |
| PSB ST-6+ 40 kg P_2_O_5_ ha ^-1^ | 28.7± 0.91**^bc^** (37.98) | 39.33± 0.96**^c^** (31.24) | 44.37± 2.14**^bc^** (35.97) |
| SEm | 4.932 | 7.712 | 11.533 |

Data were analyzed through SPSS 16.0. Duncan’s Multiple Range Test was applied. Mean ± S.E are shown.

Values in parenthesis indicate homogenous subsets at significant difference (P≤0.05). Each value is mean of three replicates, PSB: Phosphate solubilizing bacteria (1.9X 10^8^ cfu/seed). Values in brackets indicate percent increase over control.

**Supplementary table 2: Effect of PSB (ST-30, N-26, ST-6) on root length (cm) of *Cicer arietinum* L. var. PG-186**

| **Treatment** | **40 DAS** | **60 DAS** | **75 DAS** |
| --- | --- | --- | --- |
| Uninoculated control | 10.03± 0.12**^a^** | 10.67± 0.15**^a^** | 12.03± 0.23**^a^** |
| 20 kg P_2_O_5_ ha ^-1^ | 11.83± 0.28 **^b^**  (17.98) | 12.50± 0.43**^b^**(17.15) | 14.03± 0.30**^b^** (16.65) |
| 40 kg P_2_O_5_ ha ^-1^ | 14.33± 0.15**^cde^** (42.90) | 14.97± 0.27**^e^** (40.27) | 16.40± 0.31**^cd^** (36.33) |
| PSB ST-30 | 13.93± 0.24**^de^** (38.92) | 15.47± 0.53d^e^ (44.95) | 16.27± 0.39**^cd^** (35.22) |
| PSB ST-30 + 20 kg P_2_O_5_ ha ^-1^ | 15.97± 0.4**^g^** (59.19) | 17.10± 0.12**^f^** (60.26) | 18.00± 0.26**^f^** (49.63) |
| PSB ST-30 + 40 kg P_2_O_5_ ha ^-1^ | 14.57± 0.27**^ef^**  (45.23) | 15.47± 0.09**^de^** (44.95) | 16.33± 0.30**^cd^** (35.77) |
| PSB N-26 | 13.27± 0.34**^c^**  (32.27) | 14.33± 0.27**^c^** (34.33) | 16.03± 0.24**^c^** (33.28) |
| PSB N-26 + 20 kg P_2_O_5_ ha ^-1^ | 13.66± 0.26**^c^** (35.59) | 14.77± 0.26**^cd^** (38.39) | 16.93± 0.09**^de^** (40.77) |
| PSB N-26 + 40 kg P_2_O_5_ ha ^-1^ | 15.23± 0.12**^fg^** (51.88) | 15.97± 0.26**^e^** (49.64) | 17.33± 0.26**^ef^** (44.08) |
| PSB ST-6 | 13.70± 0.53**^cde^** (36.59) | 14.07± 0.37**^c^** (31.83) | 16.37±0.29**^cd^**(36.05) |
| PSB ST-6+ 20 kg P_2_O_5_ ha ^-1^ | 13.40± 0.15**^cd^** (33.60) | 14.80± 0.21**^cd^** (38.71) | 16.27± 0.19**^cd^** (35.22) |
| PSB ST-6+ 40 kg P_2_O_5_ ha ^-1^ | 14.67± 0.49**^de^** (42.24) | 15.90± 0.27**^e^** (49.02) | 16.47± 0.19**^cd^** (36.88) |
| SEm | 0.258 | 0.256 | 0.209 |

Data were analyzed through SPSS 16.0. Duncan’s Multiple Range Test was applied. Mean ± SE are shown. Values in parenthesis indicate homogenous subsets at significant difference (P≤0.05). Each value is mean of three replicates, PSB: Phosphate solubilizing bacteria (1.9X 10^8^ cfu/seed). Values in brackets indicate percent increase over control.

**Supplementary table 3: Effect of PSB (ST-30, N-26, ST-6) on fresh weight (g) of *Cicer arietinum* L. var. PG-186**

| **Treatment** | **40 DAS** | **60 DAS** | **75DAS** |
| --- | --- | --- | --- |
| Uninoculated control | 5.33± 0.18**^a^** | 10.03± 1.25**^a^** | 14.77± 0.83**^a^** |
| 20 kg P_2_O_5_ ha ^-1^ | 6.1± 0.40**^ab^** (14.45) | 15.77± 0.35**^b^** (57.18) | 18.20± 1.02**^b^** (23.22) |
| 40 kg P_2_O_5_ ha ^-1^ | 7.90± 0.55**^bc^** (48.22) | 18.43± 0.81**^c^** (83.76) | 22.57± 0.70**^cd^** (52.79) |
| PSB ST-30 | 7.90± 0.69**^bc^** (48.22) | 18.10± 0.53**^bc^** (80.43) | 22.37± 0.24**^cd^** (51.43) |
| PSB ST-30 + 20 kg P_2_O_5_ ha ^-1^ | 8.83± 0.75**^c^** (65.73) | 18.60± 0.78**^c^** (85.42) | 24.43± 0.78**^d^** (65.43) |
| PSB ST-30 + 40 kg P_2_O_5_ ha ^-1^ | 8.13± 0.29**^bc^**  (52.60) | 18.467± 0.35**^c^** (84.09) | 22.9± 0.45**^cd^** (55.04) |
| PSB N-26 | 7.33± 0.47**^abc^** (37.59) | 18.13± 0.87**^bc^** (80.77) | 21.7± 0.84**^c^** (46.92) |
| PSB N-26 + 20 kg P_2_O_5_ ha ^-1^ | 7.57± 0.98**^abc^** (41.96) | 18.30± 0.95**^c^** (82.43) | 21.87± 1.05**^cd^** (48.05) |
| PSB N-26 + 40 kg P_2_O_5_ ha ^-1^ | 7.60± 0.75**^abc^** (42.59) | 18.75± 0.69**^c^** (85.09) | 22.53± 0.88**^cd^** (52.56) |
| PSB ST-6 | 7.27± 0.97**^abc^** (36.34) | 17.53± 0.84**^bc^** (74.79) | 21.37± 0.64**^c^** (44.66) |
| PSB ST-6+ 20 kg P_2_O_5_ ha ^-1^ | 7.73± 1.05**^bc^** (45.09) | 18.03± 0.46**^bc^** (79.77) | 22.07± 1.02**^cd^** (49.40) |
| PSB ST-6+ 40 kg P_2_O_5_ ha ^-1^ | 8.03± 0.67**^bc^** (50.72) | 18.50± 0.81**^c^** (84.42) | 22.80± 0.44**^cd^** (54.37) |
| SEm | 1.455 | 1.764 | 1.821 |

Data were analyzed through SPSS 16.0. Duncan’s Multiple Range Test was applied. Mean ± S.E are shown. Values in parenthesis indicate homogenous subsets at significant difference (P≤0.05). Each value is mean of three replicates, PSB: Phosphate solubilizing bacteria (1.9X 10^8^ cfu/seed). Values in brackets indicate percent increase over control.

**Supplementary table 4: Effect of PSB (ST-30, N-26, ST-6) on dry weight (g) of *Cicer arietinum* L. var. PG-186**

| **Treatment** | **40 DAS** | **60 DAS** | **75DAS** |
| --- | --- | --- | --- |
| Uninoculated control | 1.12± 0.04**^a^** | 2.06± 0.26**^a^** | 3.15± 0.18**^a^** |
| 20 kg P_2_O_5_ ha ^-1^ | 1.28± 0.08**^ab^** (14.23) | 3.23± 0.07**^b^** (56.87) | 3.88± 0.22**^b^** (23.09) |
| 40 kg P_2_O_5_ ha ^-1^ | 1.66± 0.12**^bc^** (47.94) | 3.78± 0.17**^c^** (83.40) | 4.81± 0.15**^cd^** (52.62) |
| PSB ST-30 | 1.66± 0.15**^bc^** (47.94) | 3.71± 0.11**^bc^** (80.09) | 4.76± 0.05**^cd^** (51.27) |
| PSB ST-30 + 20 kg P_2_O_5_ ha ^-1^ | 1.85± 0.16**^c^** (65.42) | 3.81± 0.16**^c^** (85.06) | 5.21± 0.17**^d^** (65.25) |
| PSB ST-30 + 40 kg P_2_O_5_ ha ^-1^ | 1.71±0.06**^bc^** (52.31) | 3.78± 0.07**^c^**(83.73) | 4.88± 0.10**^cd^** (54.88) |
| PSB N-26 | 1.54±0.10**^abc^** (37.33) | 3.72± 0.18**^bc^** (80.42) | 4.62± 0.18**^c^** (46.76) |
| PSB N-26 + 20 kg P_2_O_5_ ha ^-1^ | 1.59± 0.21**^bc^** (41.70) | 3.75± 0.20**^c^** (82.08) | 4.66± 0.22**^cd^** (47.89) |
| PSB N-26 + 40 kg P_2_O_5_ ha ^-1^ | 1.59±0.16**^bc^** (42.32) | 3.81± 0.14**^c^** (84.73) | 4.80± 0.19**^cd^** (52.40) |
| PSB ST-6 | 1.52±0.20**^abc^** (36.08) | 3.59± 0.17**^bc^** (74.45) | 4.55± 0.14**^c^** (44.51) |
| PSB ST-6+ 20 kg P_2_O_5_ ha ^-1^ | 1.62±0.22**^bc^** (44.82) | 3.70± 0.09**^bc^** (79.42) | 4.70± 0.22**^cd^** (49.24) |
| PSB ST-6+ 40 kg P_2_O_5_ ha ^-1^ | 1.68±0.13**^bc^** (50.44) | 3.79± 0.17**^c^** (84.07) | 4.86± 0.09**^cd^** (54.20) |
| SEm | 0.059 | 0.074 | 0.083 |

Data were analyzed through SPSS 16.0. Duncan’s Multiple Range Test was applied. Mean ± S.E are shown. Values in parenthesis indicate homogenous subsets at significant difference (P≤0.05). Each value is mean of three replicates, PSB: Phosphate solubilizing bacteria (1.9x 10^8^ cfu/seed). Values in brackets indicate percent increase over control.

**Supplementary table 5: Effect of PSB (ST-30, N-26, ST-6) on nodule count (Plant-^1^) of *Cicer arietinum* L. var. PG-186**

| **Treatment** | **40 DAS** | **60 DAS** | **75DAS** |
| --- | --- | --- | --- |
| Uninoculated control | 6.4± 1.01**^a^** | 8.8± 0.70**^a^** | 7.8± 1.14**^a^** |
| 20 kg P_2_O_5_ ha ^-1^ | 8.4± 1.01**^ab^** (31.25) | 10.33± 1.19**^ab^** (17.42) | 9.53± 1.23**^ab^** (22.22) |
| 40 kg P_2_O_5_ ha ^-1^ | 10.2± 0.69**^bc^** (59.38) | 12.4± 0.76**^bc^** (40.91) | 12± 0.58**^bc^** (53.85) |
| PSB ST-30 | 13± 0.92**^cd^** (103.13) | 15± 0.83**^de^** (70.45) | 13.73± 0.81**^cd^** (76.07) |
| PSB ST-30 + 20 kg P_2_O_5_ ha ^-1^ | 13.93± 0.82**^d^** (117.71) | 16.53± 0.35**^e^** (87.88) | 15.27± 0.59**^d^** (95.73) |
| PSB ST-30 + 40 kg P_2_O_5_ ha ^-1^ | 13.27± 0.48**^cd^** (107.29) | 15.13± 1.07**^de^** (71.97) | 14.13± 0.52**^cd^** (81.20) |
| PSB N-26 | 11.67± 1.01**^bcd^** (82.29) | 13.53± 0.74**^cd^** (53.79) | 12.27± 0.87**^bc^** (57.26) |
| PSB N-26 + 20 kg P_2_O_5_ ha ^-1^ | 11.53± 0.93**^bcd^** (80.21) | 14± 0.50**^cd^** (59.09) | 12.33± 1.23**^bc^** (58.12) |
| PSB N-26 + 40 kg P_2_O_5_ ha ^-1^ | 10.53± 1.23**^bcd^** (64.58) | 13.5± 0.29**^cd^** (53.41) | 12.2± 1.29**^bc^** (56.41) |
| PSB ST-6 | 10.6± 1.25**^bcd^** (65.63) | 13.2± 0.42**^cd^** (50.00) | 11.7± 0.46**^bc^** (50.00) |
| PSB ST-6+ 20 kg P_2_O_5_ ha ^-1^ | 10.73± 1.38**^bcd^**  (67.71) | 13.53± 0.87**^cd^** (53.79) | 11.6± 0.46**^bc^** (48.72) |
| PSB ST-6+ 40 kg P_2_O_5_ ha ^-1^ | 10.67± 1.23**^bcd^** (66.67) | 13.33± 0.55**^cd^** (51.52) | 12.03± 1.05**^bc^** (54.27) |
| SEm | 3.158 | 1.638 | 2.472 |

Data were analyzed through SPSS 16.0. Duncan’s Multiple Range Test was applied. Mean ± S.E are shown. Values in parenthesis indicate homogenous subsets at significant difference (P≤0.05). Each value is mean of three replicates, PSB: Phosphate solubilizing bacteria (1.9X 10^8^ cfu/seed). Values in brackets indicate percent increase over control.

**Supplementary table 6: Effect of PSB (ST-30, N-26, ST-6) on nodule fresh weight Plant-^1^ (mg) of *Cicer arieinum* L. var. PG-186**

| **Treatment** | **40 DAS** | **60 DAS** | **75DAS** |
| --- | --- | --- | --- |
| Uninoculated control | 321.53± 12.73**^a^** | 402.83± 16.30**^a^** | 353.77± 11.44**^a^** |
| 20 kg P_2_O_5_ ha ^-1^ | 425.43± 15.96**^b^** (32.3) | 568.70± 41.03**^b^** (41.2) | 479.13± 38.95**^b^** (35.4) |
| 40 kg P_2_O_5_ ha ^-1^ | 635.93± 14.88**^cde^** (97.8) | 769.50± 25.36**^cde^** (91.0) | 667.07± 16.86**^cde^** (88.6) |
| PSB ST-30 | 661.33± 13.39**^efg^** (105.7) | 829.10± 14.64**^ef^** (105.8) | 783.80± 9.33**^g^** (121.6) |
| PSB ST-30 + 20 kg P_2_O_5_ ha ^-1^ | 737.60± 8.85**^fg^** (129.4) | 909.80± 44.63**^f^** (125.9) | 790.60± 51.00**^g^** (123.5) |
| PSB ST-30 + 40 kg P_2_O_5_ ha ^-1^ | 697.67± 7.98**^g^**  (117.0) | 831.17± 12.37**^ef^** (106.3) | 762.70± 11.44**^fg^** (115.6) |
| PSB N-26 | 693.10± 11.02**^h^** (115.6) | 753.30± 12.86**^cde^** (87.0) | 707.77± 18.27**^def^** (100.1) |
| PSB N-26 + 20 kg P_2_O_5_ ha ^-1^ | 656.27± 13.95**^defg^** (104.1) | 806.00± 33.46**^de^** (100.1) | 724.17± 22.46**^efg^** (104.7) |
| PSB N-26 + 40 kg P_2_O_5_ ha ^-1^ | 651.90± 9.56**^cdef^**  (102.7) | 789.77± 11.49**^cde^** (96.1) | 696.53± 11.16**^def^** (96.9) |
| PSB ST-6 | 613.03± 14.73**^cd^** (90.7) | 711.70± 26.09**^c^** (76.7) | 620.50± 17.20**^c^** (75.4) |
| PSB ST-6+ 20 kg P_2_O_5_ ha ^-1^ | 649.03± 20.28**^cde^** (101.9) | 760.13± 30.42**^cde^** (88.7) | 668.07± 5.18**^cde^** (88.8) |
| PSB ST-6+ 40 kg P_2_O_5_ ha ^-1^ | 608.30± 15.46**^c^** (89.2) | 727.93± 29.88**^cd^** (80.7) | 636.37± 13.56**^cd^** (79.9) |
| SEm | 558.430 | 2216.643 | 1555.077 |

Data were analyzed through SPSS 16.0. Duncan’s Multiple Range Test was applied. Mean ± S.E are shown. Values in paranthesis indicate homogenous subsets at significant difference (P≤0.05). Each value is mean of three replicates, PSB: Phosphate solubilizing bacteria (1.9X 10^8^ cfu/seed). Values in brackets indicate percent increase over control.

**Supplementary table 7: Effect of PSB (ST-30, N-26, ST-6) on nodule dry weight Plant-^1^ (mg) of *Cicer arietinum* L. var. PG-186**

| **Treatment** | **40 DAS** | **60 DAS** | **75DAS** |
| --- | --- | --- | --- |
| Uninoculated control | 68.22± 2.70**^a^** | 86.21± 3.49**^a^** | 75.34± 2.45^a^ |
| 20 kg P_2_O_5_ ha ^-1^ | 90.27± 3.39**^b^** (32.3) | 123.41± 7.16**^b^** (43.2) | 102.70± 8.27**^b^**  (36.3) |
| 40 kg P_2_O_5_ ha ^-1^ | 134.93± 3.16**^cde^** (97.8) | 164.72± 5.34**^cde^** (91.1) | 142.49± 3.87**^cde^** (89.1) |
| PSB ST-30 | 140.32± 2.84**^efg^** (105.7) | 177.39± 3.13**^ef^** (105.8) | 166.87± 1.99**^g^**  (121.5) |
| PSB ST-30 + 20 kg P_2_O_5_ ha ^-1^ | 156.50± 1.88**^h^** (129.4) | 194.65± 9.55**^f^** (125.8) | 168.43± 10.79**^g^**  (123.6) |
| PSB ST-30 + 40 kg P_2_O_5_ ha ^-1^ | 148.03± 1.69**^g^** (117.0) | 178.31± 3.12**^ef^** (106.8) | 165.00± 2.26**^fg^**  (119.0) |
| PSB N-26 | 147.06± 2.34**^fg^** (115.6) | 161.56± 3.11**^cde^** (87.4) | 150.93± 3.72**^def^** (100.3) |
| PSB N-26 + 20 kg P_2_O_5_ ha ^-1^ | 139.25± 2.96**^defg^** (104.1) | 172.44± 7.16**^de^** (100.0) | 154.47± 4.70**^efg^** (105.0) |
| PSB N-26 + 40 kg P_2_O_5_ ha ^-1^ | 138.32± 2.03**^cdef^** (102.7) | 168.97± 2.46**^cde^** (96.0) | 148.29± 2.38**^de^**  (96.8) |
| PSB ST-6 | 130.07± 3.12**^cd^** (90.7) | 152.79± 6.02**^c^** (77.2) | 132.11± 3.66**^c^** (75.4) |
| PSB ST-6+ 20 kg P_2_O_5_ ha ^-1^ | 137.71± 4.30**^cde^** (101.9) | 163.03± 6.25**^cde^** (89.1) | 142.31± 1.18**^cde^** (88.9) |
| PSB ST-6+ 40 kg P_2_O_5_ ha ^-1^ | 129.07± 3.28**^c^** (89.2) | 156.08± 6.38**^cd^** (81.1) | 135.49± 2.90**^cd^** (79.9) |
| SEm | 25.154 | 96.459 | 69.974 |

Data were analyzed through SPSS 16.0. Duncan’s Multiple Range Test was applied. Mean ± S.E are shown. Values in parenthesis indicate homogenous subsets at significant difference (P≤0.05). Each value is mean of three replicates, PSB: Phosphate solubilizing bacteria (1.9X 10^8^ cfu/seed). Values in brackets indicate percent increase over control.

**Supplementary table 8: Effect of PSB (ST-30, N-26, ST-6) on chlorophyll content (mg g^-1^ fresh weight) of *Cicer arietinum* L. var. PG-186**

| **Treatment** | **40 DAS** | **60 DAS** | **75DAS** |
| --- | --- | --- | --- |
| Uninoculated control | 2.12± 0.01**^a^** | 2.64± 0.06**^a^** | 2.21± 0.08^a^ |
| 20 kg P_2_O_5_ ha ^-1^ | 2.48± 0.08**^ab^** (16.74) | 3.00± 0.12**^b^** (13.89) | 2.75± 0.03**^b^** (24.43) |
| 40 kg P_2_O_5_ ha ^-1^ | 2.63± 0.02**^bc^** (23.68) | 3.06± 0.15**^b^** (16.09) | 2.90± 0.04**^bc^** (31.39) |
| PSB ST-30 | 2.68± 0.04**^bc^** (26.08) | 3.09± 0.14**^b^** (17.29) | 2.90± 0.09**^bc^** (31.36) |
| PSB ST-30 + 20 kg P_2_O_5_ ha ^-1^ | 2.95± 0.03**^c^** (38.76) | 3.26± 0.07**^b^** (23.75) | 3.00± 0.04**^cd^** (35.69) |
| PSB ST-30 + 40 kg P_2_O_5_ ha ^-1^ | 2.84± 0.07**^bc^** (33.55) | 3.15± 0.07**^b^** (19.60) | 3.08± 0.04**^d^** (39.37) |
| PSB N-26 | 2.52± 0.08**^abc^** (18.63) | 3.00± 0.06**^b^** (13.68) | 2.86± 0.04**^bc^** (29.45) |
| PSB N-26 + 20 kg P_2_O_5_ ha ^-1^ | 2.72± 0.04**^bc^** (28.07) | 3.05± 0.09**^b^** (15.58) | 2.99± 0.02**^bcd^** (35.53) |
| PSB N-26 + 40 kg P_2_O_5_ ha ^-1^ | 2.66± 0.03**^bc^** (25.28) | 3.00± 0.04**^b^** (13.72) | 2.92± 0.01**^bcd^** (32.40) |
| PSB ST-6 | 2.52± 0.04**^abc^** (18.48) | 3.06± 0.12**^b^** (16.13) | 2.91± 0.01**^bc^** (31.72) |
| PSB ST-6+ 20 kg P_2_O_5_ ha ^-1^ | 2.72± 0.02**^bc^** (28.07) | 3.18± 0.15**^b^** (20.40) | 2.94± 0.02**^bcd^** (33.06) |
| PSB ST-6+ 40 kg P_2_O_5_ ha ^-1^ | 2.73± 0.05**^bc^** (28.77) | 3.06± 0.04**^b^** (16.23) | 2.92± 0.03**^bcd^** (32.35) |
| SEM | 0.052 | 0.030 | 0.022 |

Data were analyzed through SPSS 16.0. Duncan’s Multiple Range Test was applied. Mean ± S.E are shown. Values in parenthesis indicate homogenous subsets at significant difference (P≤0.05). Each value is mean of three replicates, PSB: Phosphate solubilizing bacteria (1.9X 10^8^ cfu/seed). Values in brackets indicate percent increase over control.

**Supplementary table 9: Effect of PSB (ST-30, N-26, ST-6) on nitrate reductase activity (µ mol NO_2_ ^-^ g^-1^ fresh weight) of *Cicer arietinum* L. var. PG-186**

| **Treatment** | **40 DAS** | **60 DAS** | **75DAS** |
| --- | --- | --- | --- |
| Uninoculated control | 0.39± 0.035**^a^** | 0.897± 0.14**^a^** | 1.03± 0.043**^a^** |
| 20 kg P_2_O_5_ ha ^-1^ | 0.50± 0.098**^ab^** (27.35) | 1.153± 0.05**^b^** (28.62) | 1.12± 0.037**^a^** (8.85) |
| 40 kg P_2_O_5_ ha ^-1^ | 0.58± 0.049**^bc^**(48.72) | 1.213± 0.03**^bc^** (35.31) | 1.19± 0.064**^ab^** (15.68) |
| PSB ST-30 | 0.67± 0.058**^bc^** (70.94) | 1.524± 0.06**^ef^** (69.96) | 1.45± 0.060**^b^** (40.91) |
| PSB ST-30 + 20 kg P_2_O_5_ ha ^-1^ | 0.65± 0.026**^bc^**(66.67) | 1.527± 0.06**^ef^** (70.25) | 1.44± 0.042**^b^** (40.90) |
| PSB ST-30 + 40 kg P_2_O_5_ ha ^-1^ | 0.68± 0.032**^c^** (75.25) | 1.520± 0.04**^ef^** (69.51) | 1.45± 0.031**^b^** (40.91) |
| PSB N-26 | 0.70± 0.015**^c^** (81.20) | 1.527± 0.05**^ef^** (70.25) | 1.45± 0.061**^b^** (41.21) |
| PSB N-26 + 20 kg P_2_O_5_ ha ^-1^ | 0.73± 0.016**^c^** (88.03) | 1.553± 0.05**^ef^** (73.23) | 1.46± 0.061**^b^** (41.63) |
| PSB N-26 + 40 kg P_2_O_5_ ha ^-1^ | 0.75± 0.015**^c^** (91.45) | 1.560± 0.06**^f^** (73.97) | 1.47± 0.064**^b^** (42.60) |
| PSB ST-6 | 0.61± 0.078**^bc^**(57.26) | 1.427± 0.13**^ef^** (59.10) | 1.43± 0.065**^b^** (38.97) |
| PSB ST-6+ 20 kg P_2_O_5_ ha ^-1^ | 0.64± 0.064**^bc^** (63.25) | 1.350± 0.02**^cd^** (50.51) | 1.20± 0.232**^ab^** (16.29) |
| PSB ST-6+ 40 kg P_2_O_5_ ha ^-1^ | 0.65± 0.050**^bc^** (66.67) | 1.353± 0.02**^cd^** (50.92) | 1.14± 0.440**^a^** (11.11) |
| SEM | 0.008 | 0.011 | 0.021 |

Data were analyzed through SPSS 16.0. Duncan’s Multiple Range Test was applied. Mean ± S.E are shown. Values in parenthesis indicate homogenous subsets at significant difference (P≤0.05). Each value is mean of three replicates, PSB: Phosphate solubilizing bacteria (1.9X 10^8^ cfu/seed). Values in brackets indicate percent increase over control.

**Supplementary table 10: Effect of PSB (ST-30, N-26, ST-6) on Plant P content (mg g^-1^ dry weight) of *Cicer arietinum* L. var. PG-186**

| **Treatment** | **40 DAS** | **60 DAS** | **75DAS** |
| --- | --- | --- | --- |
| Uninoculated control | 3.03± 0.46**^a^** | 3.35± 0.27**^a^** | 3.17± 0.09**^a^** |
| 20 kg P_2_O_5_ ha ^-1^ | 3.77± 0.28**^ab^** (24.18) | 3.94± 0.09**^a^** (17.40) | 3.75± 0.06**^ab^** (18.42) |
| 40 kg P_2_O_5_ ha ^-1^ | 4.90± 0.26**^bcde^** (61.55) | 5.23± 0.06**^b^** (55.87) | 4.80± 0.15**^bc^** (51.59) |
| PSB ST-30 | 4.83± 0.58**^bcde^** (59.35) | 6.13± 0.58**^bc^** (82.90) | 6.03± 0.46**^cde^** (90.54) |
| PSB ST-30 + 20 kg P_2_O_5_ ha ^-1^ | 5.77± 0.35**^e^** (90.12) | 7.10± 0.23**^c^** (111.73) | 7.03± 0.52**^e^** (122.13) |
| PSB ST-30 + 40 kg P_2_O_5_ ha ^-1^ | 5.70± 0.26**^de^** (87.92) | 6.93± 0.32**^c^** (106.76) | 6.80± 0.40**^de^** (114.76) |
| PSB N-26 | 4.53± 0.23**^bcd^** (49.46) | 5.53± 0.41**^b^** (65.01) | 5.47± 0.64**^cd^** (72.65) |
| PSB N-26 + 20 kg P_2_O_5_ ha ^-1^ | 4.73± 0.23**^bcde^** (56.05) | 5.90± 0.45**^bc^** (75.95) | 5.73± 0.49**^cde^** (81.07) |
| PSB N-26 + 40 kg P_2_O_5_ ha ^-1^ | 4.93± 0.35**^cde^** (62.65) | 6.23± 0.32**^bc^** (85.89) | 6.13± 0.49**^cde^** (93.70) |
| PSB ST-6 | 4.43± 0.29**^bc^** (46.16) | 5.43± 0.49**^b^** (62.03) | 5.27± 0.46**^c^** (66.33) |
| PSB ST-6+ 20 kg P_2_O_5_ ha ^-1^ | 5.03± 0.32**^cde^** (65.94) | 5.80± 0.64**^bc^** (72.96) | 5.63± 0.20**^cd^** (77.91) |
| PSB ST-6+ 40 kg P_2_O_5_ ha ^-1^ | 5.13± 0.39**^cde^** (69.24) | 6.13± 0.49**^bc^** (82.90) | 5.97± 0.43**^cde^** (88.44) |
| SEm | 0.367 | 0.482 | 0.503 |

Data were analyzed through SPSS 16.0. Duncan’s Multiple Range Test was applied. Mean ± S.E are shown. Values in parenthesis indicate homogenous subsets at significant difference (P≤0.05). Each value is mean of three replicates, PSB: Phosphate solubilizing bacteria (1.9X 10^8^ cfu/seed). Values in brackets indicate percent increase over control.
